# Research on detection methods of related substances and degradation products of the antitumor drug selpercatinib

**DOI:** 10.3389/fchem.2024.1534132

**Published:** 2025-01-13

**Authors:** Jingjing Xiang, Liangliang Cai, Qin Wang, Yonghong Zhu, Yong Han

**Affiliations:** ^1^ Department of Pharmacy, Affiliated Nantong Hospital of Shanghai University (The Sixth People’s Hospital of Nantong), Nantong, China; ^2^ Department of Pharmacy, Affiliated Hospital of Nantong University, Nantong, China; ^3^ Department of Oncology, Shanghai General Hospital, School of Medicine, Shanghai Jiao Tong University, Shanghai, China

**Keywords:** selpercatinib, related substances, degradation products, method development, method validation, liquid chromatography

## Abstract

**Background:**

Selpercatinib, a selective RET kinase inhibitor, is approved for treating various cancers with RET gene mutations such as RET-rearranged thyroid cancer and non-small cell lung cancer. The presence of process-related and degradation impurities in its active pharmaceutical ingredient (API) can significantly affect its safety and effectiveness. However, research on detecting these impurities is limited.

**Methods:**

This study developed and systematically validated a High-Performance Liquid Chromatography (HPLC) method for identifying selpercatinib and its related impurities. The method utilized a 4.6 mm × 250 mm chromatographic column with 5 μm particles, employing a flow rate of 1.0 mL/min, a detection wavelength of 235 nm, an injection volume of 10 μL, and a column temperature of 35°C. Mobile phase A was composed of a 9:1 ratio of water to acetonitrile, with the aqueous component adjusted to pH 2.5 and containing 2 mM potassium dihydrogen phosphate (KH_2_PO_4_) and 0.4% triethylamine. Mobile phase B was pure acetonitrile. The gradient elution program was as follows: 0–2 min, 5%B; 2–15 min, 5% to 15%B; 15–30 min, 15% to 35%B; 30–35 min, 35% to 45%B; 35–36 min, 45% to 5%B; 36–45 min, 5%B.

**Results:**

The chromatographic method established in this study was validated according to the ICH Q2 (R1) guidelines. The developed HPLC method demonstrated excellent specificity, sensitivity, stability, linearity, precision, accuracy, and robustness. It efficiently separated the impurities present in selpercatinib, thereby confirming the method’s efficacy in ensuring the purity and quality of the drug.

**Conclusion:**

The chromatographic method established in this study can be used for the detection of selpercatinib and its impurities, providing significant reference value for the quality research of selpercatinib bulk drug and its preparations, and ensuring the safety of medication for patients.

## 1 Introduction

Lung cancer is among the most prevalent malignant tumors globally, representing a significant threat to human health and presenting a major challenge to public health (Bade and Cruz, 2020; Zhang et al., 2023). According to the most recent statistics from the International Agency for Research on Cancer (IARC) of the World Health Organization (WHO), lung cancer ranked first in both incidence and mortality rates among 36 types of cancer across 185 countries in 2022, underscoring its extensive impact and peril (Bray et al., 2024). Among the various subtypes of lung cancer, non-small cell lung cancer (NSCLC) is the most prevalent, comprising approximately 85%–90% of all lung cancer cases (Pretelli et al., 2023; Restrepo et al., 2024). Consequently, research directed toward non-small cell lung cancer has emerged as a critical area of investigation within the field of oncology.

Currently, the primary treatment strategies for non-small cell lung cancer include surgical intervention, radiotherapy, chemotherapy, immunotherapy, and targeted therapy (Visa et al., 2024). Surgical treatment is generally applicable to patients diagnosed at an early stage, while radiotherapy is primarily used to control the growth of localized tumors. Although chemotherapy is widely utilized in advanced cases, its detrimental effects on normal cells can lead to severe side effects, significantly reducing the patient’s quality of life (Liu et al., 2020; Purushothaman et al., 2020). In contrast, targeted therapy is increasingly emphasized in the medical community due to its high specificity. Targeted therapy is designed to target specific molecules or genetic mutations within cancer cells, and compared to traditional chemotherapy, its advantages include enhanced treatment efficacy, improved patient tolerance, reduced recurrence rates, and minimized side effects (Crintea et al., 2023; Cui et al., 2021). Common targeted drugs currently include EGFR inhibitors, ALK inhibitors, KRAS inhibitors, and RET inhibitors (Cooper et al., 2022; Griesinger et al., 2022; Nakajima et al., 2022; Shi et al., 2022).

RET is a transmembrane glycoprotein receptor tyrosine kinase encoded by the RET proto-oncogene, which is located on chromosome 10 (Servetto et al., 2022). This protein comprises three components: an extracellular domain, a transmembrane domain, and an intracellular tyrosine kinase domain responsible for catalytic activity. This structural configuration of the transmembrane protein facilitates the effective transmission of signals between the cell’s interior and exterior. When pathogenic mutations arise in the RET gene (e.g., point mutations, gene rearrangements, fusions), RET proteins may exhibit abnormal activities that transmit aberrant signals, resulting in altered cellular behaviors such as growth, survival, invasion, and metastasis. Prolonged signaling may ultimately contribute to tumor development and progression. RET mutations have been identified in various cancer types, including medullary thyroid cancer, papillary thyroid cancer, and lung cancer—particularly non-small cell lung cancer, where RET rearrangements account for approximately 1%–2% of cases (Thein et al., 2021; Tiurin et al., 2023). At present, the treatment of lung cancers exhibiting RET alterations primarily concentrates on the development of targeted therapies. For instance, RET inhibitors, including pralsetinib and selpercatinib (also referred to as LOXO-292), have been approved for the treatment of RET fusion-positive or mutant non-small cell lung cancer (Griesinger et al., 2022; Wright, 2020).

Selpercatinib is an orally administered selective RET (rearranged during transfection) kinase inhibitor that effectively inhibits the activity of RET and its downstream phosphorylated molecules, thereby blocking cell proliferation induced by RET gene mutations (Majeed et al., 2021). Compared to previously approved multi-kinase inhibitors, selpercatinib exhibits significantly enhanced selectivity for RET, enabling more precise inhibition of cell proliferation associated with RET gene mutations. Furthermore, selpercatinib can inhibit both primary and secondary mutations, positioning it as a potential solution to address clinical drug resistance.

Currently, research on selpercatinib primarily focuses on its clinical efficacy and safety in the treatment of non-small cell lung cancer (Cheng et al., 2024; Drilon et al., 2023). At the same time, studies have employed high-performance liquid chromatography-tandem mass spectrometry (HPLC-MS/MS) to measure plasma drug concentrations in patients administered selpercatinib (Gulikers et al., 2023). Furthermore, researchers, such as Katta and Divya, have primarily focused on studying the impurities produced during forced degradation of selpercatinib and identifying their structures using liquid chromatography-mass spectrometry (LC-MS). The high-performance liquid chromatography (HPLC) method they established is only used for detecting degradation impurities with higher contents, while neglecting the separation of degradation products with unknown structures and process impurities (Katta and Thakre, 2024; Divya et al., 2024). Notably, there are currently relatively few studies on the detection of process impurities and their degradation impurities (including both known and unknown structures) in selpercatinib active pharmaceutical ingredients (API) using HPLC. Related substances in the API, including process-related impurities and degradation products, directly affect the safety and efficacy of the drug and are critical factors in pharmaceutical production and quality control. Consequently, the development of analytical methods for detecting selpercatinib and its impurities is of paramount importance.

HPLC is a widely utilized analytical technique known for its high sensitivity, high selectivity, broad applicability, accurate quantification capability, and cost-effectiveness. Currently, HPLC technology remains a commonly used method for detecting related substances in active pharmaceutical ingredients (API) (Ni et al., 2022; Zhu et al., 2024). Moreover, the majority of pharmacopoeias globally, including the United States Pharmacopeia, European Pharmacopoeia, Chinese Pharmacopoeia, Japanese Pharmacopoeia, and British Pharmacopoeia, designate liquid chromatography as the preferred analytical method for detecting related substances in APIs. At present, research regarding the detection of related substances in the API of selpercatinib remains limited. Given the potential impact of process-related impurities and degradation products in the API on the efficacy and safety of selpercatinib, there is an urgent need to develop a method capable of simultaneously detecting both process-related impurities and degradation products in selpercatinib.

In this study, we successfully established and implemented a validated reversed-phase high-performance liquid chromatography (RP-HPLC) method for the detection of related substances in selpercatinib. This method is characterized by its simplicity, sensitivity, accuracy, and robustness, particularly in effectively separating compounds associated with selpercatinib, including impurities and degradation products generated during the manufacturing process (imp-A, imp-B, imp-C, imp-D). Subsequently, we conducted a comprehensive evaluation of the method to assess its specificity, sensitivity, solution stability, linearity, precision, accuracy, and robustness. Furthermore, we evaluated key parameters such as the limit of quantitation (LOQ), limit of detection (LOD), linearity, and recovery of the RP-HPLC method. In conclusion, the RP-HPLC method established in this study is suitable for the detection of related substances in selpercatinib, providing essential assurance regarding the safety and efficacy of the drug.

## 2 Materials and methods

### 2.1 Chemicals and reagents

Selpercatinib and its known impurities (imp-A, imp-B, imp-C and imp-D) were sourced from Aladdin Chemical Reagents Co., LTD. (Beijing, China). HPLC-grade acetonitrile (ACN) and methanol (MeOH) were obtained from Anaqua Chemicals Supply Co., Ltd. (Wilmington, United States).

### 2.2 Instruments

During the method development and validation process, two primary pieces of equipment were utilized: an Agilent 1200 HPLC system equipped with a UV detector, and a Shimadzu LC-20AD system with a Photodiode Array Detector.

### 2.3 HPLC conditions

Selpercatinib and its impurities were separated using a chromatographic column (4.6 mm × 250 mm, 5 μm) with a flow rate of 1.0 mL/min. The experiment was conducted with a detection wavelength of 235 nm, an injection volume of 10 μL, and a column temperature of 35°C. Mobile phase A was composed of water and acetonitrile in a 9:1 ratio, with the aqueous component adjusted to pH 2.5, containing 2 mM potassium dihydrogen phosphate (KH_2_PO_4_) and 0.4% triethylamine. In contrast, mobile phase B consisted solely of ACN. The gradient elution program was as follows: 0–2 min, 5%B → 5%B; 2–15 min, 5%B → 15%B; 15–30 min, 15%B → 35%B; 30–35 min, 35%B → 45%B; 35–36 min, 45%B → 5%B; 36–45 min, 5%B → 5%B.

### 2.4 Preparation of stock solution

#### 2.4.1 Preparation of selpercatinib stock solution

Approximately 10 mg of selpercatinib was accurately weighed and transferred into a 20 mL volumetric flask. Subsequently, 15 mL of a 50% aqueous methanol solution was added to the flask and ultrasonic treatment was employed to dissolve the selpercatinib. The solution was then diluted with the 50% methanol solution to the calibration line to obtain the stock solution of selpercatinib.

#### 2.4.2 Preparation of selpercatinib–related substance stock solutions

Approximately 10 mg of each of the four impurities of selpercatinib (Impurities A, B, C, D) was accurately weighed and placed into five separate 20 mL volumetric flasks. Subsequently, 15 mL of 50% methanol aqueous solution was added to each flask, and the impurities were dissolved using ultrasonication. The solutions were then diluted to the calibration mark with the 50% methanol aqueous solution to prepare the stock solutions of each impurity.

### 2.5 Preparation of mixed solutions and system suitability solutions

Selpercatinib, weighting precisely 10 mg, was accurately weighed and transferred it into a 20 mL volumetric flask. Then, the compound was dissolved in 10 mL of methanol using ultrasonication to ensure complete dissolution. Subsequently, 10 mL of 1 M hydrochloric acid (HCl) solution was added. Following this, the resulting mixture was heated at 80°C for 3 h. After the reaction is complete, the mixture was neutralized with 1 M sodium hydroxide (NaOH) solution. Next, the mixture was diluted with 50% aqueous methanol to a total volume of 20 mL, thereby obtaining the acidic degradation products.

In the same way, a further 10 mg of selpercatinib was precisely measured and placed into a different 20 mL volumetric flask. The compound was dissolved in 10 mL of methanol via ultrasonication. After achieving complete dissolution, 10 mL of 15% hydrogen peroxide solution was added. The flask was maintained in a 70°C water bath and maintain the reaction for 3 h. Upon completion of the reaction, manganese dioxide was added to terminate the reaction, and the mixture was subsequently filtered to remove the manganese dioxide. The system suitability solution was then prepared by mixing equal proportions of the aforementioned acid degradation solution and oxidative degradation solution.

### 2.6 Preparation of the sample solution

To prepare the sample solution, precisely 10 mg of selpercatinib was weighed and transferred into a 20 mL volumetric flask. Subsequently, 15 mL of a 50% aqueous methanol solution was measured and employed to dissolve the selpercatinib through ultrasonic treatment. Following this, the solution was further diluted with 50% aqueous methanol to the mark, resulting in a sample solution with a concentration of approximately 0.5 mg/mL.

## 3 Results and discussion

### 3.1 Method development

**FIGURE 1:**
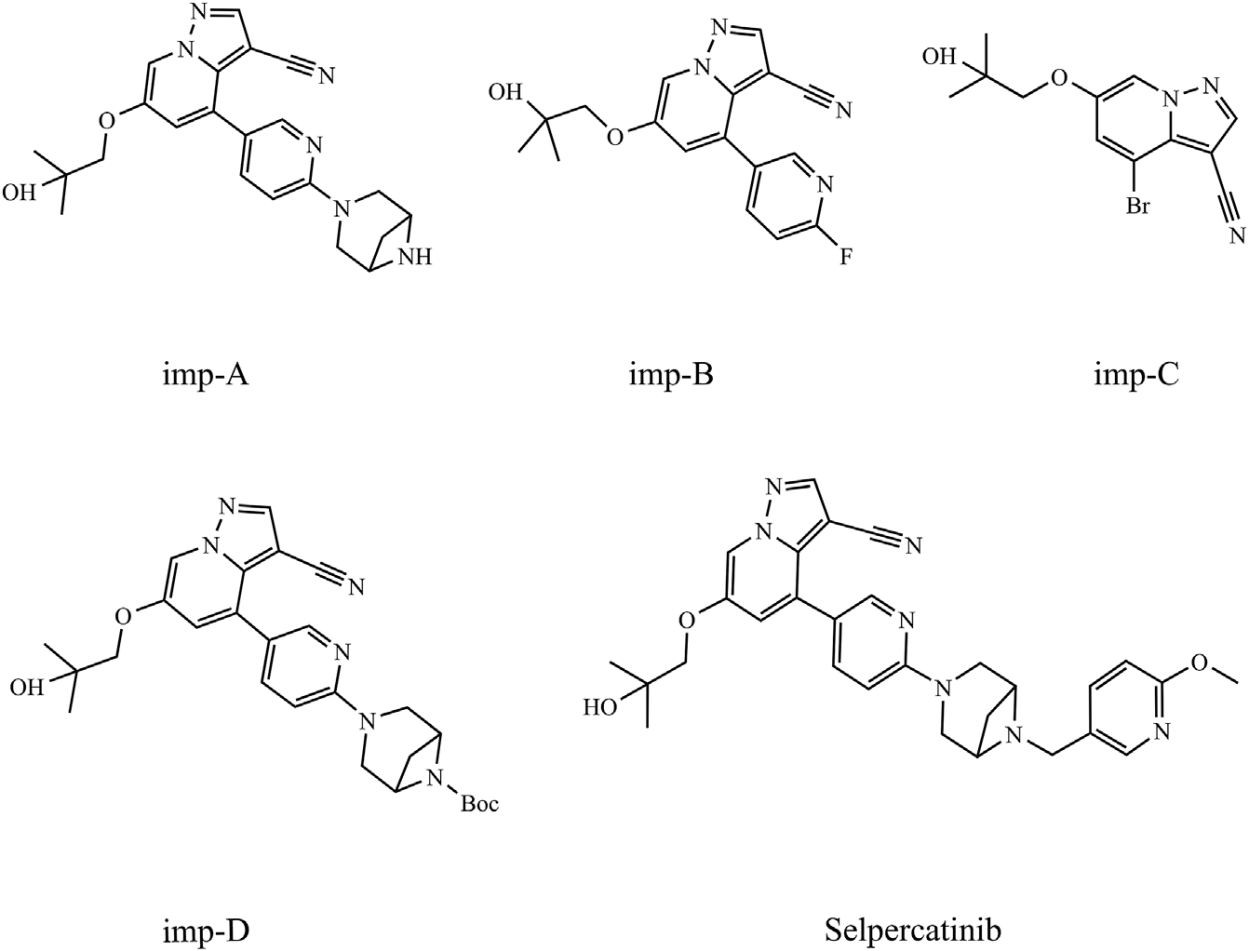
Chemical structures of selpercatinib and its known impurities.

The synthetic route for selpercatinib is derived from the patent (WO 2018/071447A1). As illustrated in Supplementary Figure S1, the synthesis process comprises six primary steps, resulting in five intermediates. Based on the intermediates generated during the synthesis of selpercatinib and the results of their degradation experiments, this study primarily investigates four impurities, namely imp-A, imp-B, imp-C, and imp-D, whose structures are depicted in Figure 1.

It is noteworthy that during the production, storage, and transportation of selpercatinib, other degradation impurities, including unknown impurities, may be generated. The formation of these unknown impurities can be studied through forced degradation testing of the selpercatinib. Accordingly, this study aims to establish a RP-HPLC method for the simultaneous detection of known impurities and potential unknown impurities in selpercatinib.

**FIGURE 2:**
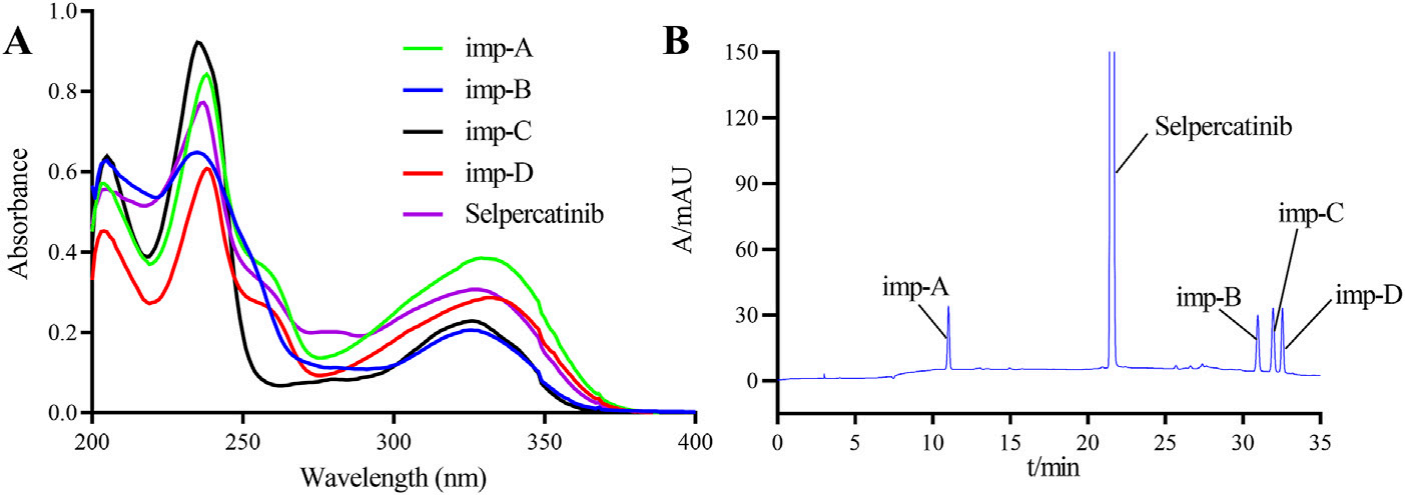
Ultraviolet spectrogram **(A)** and chromatogram **(B)** of selpercatinib and its known impurities.

To establish this detection method, we investigated the effects of detection wavelength, mobile phase composition, and elution mode on the separation of samples. To determine the optimal detection wavelength, the standard stock solution of selpercatinib and the stock solutions of its known impurities were diluted 50-fold, and subsequently scanned and analyzed using a UV-Vis spectrophotometer in the wavelength range of 200–400 nm. Figure 2A illustrates the UV spectra of selpercatinib and its known impurities. The results indicated that selpercatinib and its impurities exhibited two primary absorption peaks within the 200–400 nm range, located at 235 nm and 330 nm, respectively. Among these, at a wavelength of 235 nm, selpercatinib and its impurities exhibited stronger absorption and higher response signals, therefore, we determined the optimal detection wavelength to be 235 nm.

Given the high number of impurities present in the system suitability solution and the existence of difficult-to-separate impurity peak pairs, this solution was selected as the target for method development. Considering the presence of basic groups such as secondary and tertiary amines in the structures of selpercatinib and its impurities, the samples exhibit weakly basic characteristics. Based on this, we prioritized the use of phosphate solutions with buffering capabilities when selecting the mobile phase. However, during preliminary experiments, we observed tailing of the chromatographic peaks. To mitigate this phenomenon, triethylamine was added to the buffer solution. After multiple trials and optimizations, we ultimately determined the following mobile phase conditions: Mobile phase A consists 2 mmol/L KH_2_PO_4_ (containing 0.4% triethylamine, pH adjusted to 2.5), and contains 10% of ACN. Mobile phase B consists of ACN.

**FIGURE 3:**
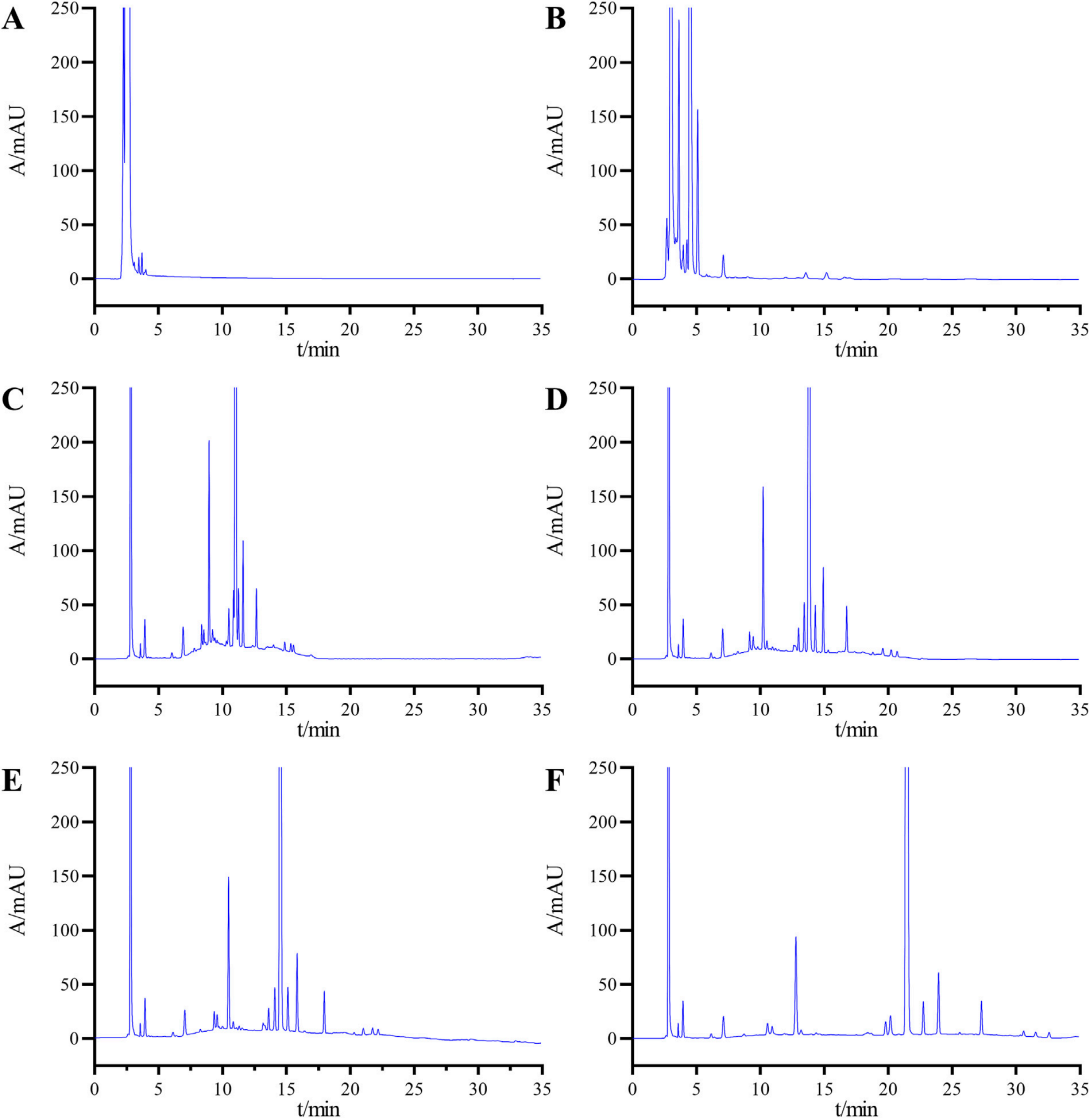
Chromatograms obtained during the optimization of the HPLLC conditions: mobile phase B and mobile phase A at ratios of 70:30 **(A)** and 30:70 **(B)**; **(C)** gradient elution condition 1; **(D)** gradient elution condition 2; **(E)** gradient elution Condition 3, and **(F)** final determined gradient elution condition.

In selecting the elution method, we initially attempted an isocratic elution approach, specifically testing with mobile phase B and mobile phase A at ratios of 70:30 and 30:70, respectively. However, the chromatograms (Figures 3A,B) revealed significant overlap among the impurity peaks, and the resolution between impurities and the main peak did not meet the specified standard requirements. Given the high number of impurities with similar polarities in the system suitability solution, the isocratic elution method was found to be insufficient for achieving the desired separation effect. Consequently, we transitioned to a gradient elution scheme for further optimization attempts.

Initially, we applied Gradient Condition 1 with the following time and composition adjustments: 0–2 min at 10%B, 2–20 min transitioning from 10%B to 90%B, 20–30 min maintaining 90%B, 30–30.1 min reverting from 90%B to 10%B, and 30.1–40 min at 10%B. As shown in Figure 3C, under this condition, the minimum resolution between impurities was 1.15, and the resolution between impurities and the main peak was 1.85, both of which failed to meet the specified requirements.

Subsequently, we further optimized the elution process by reducing the gradient change rate. We analyzed the samples using two novel gradient conditions: Gradient Condition 2, which follows the sequence 0–2 min (10%B), 2–30 min (10%B to 90%B), 30–35 min (90%B), 35–35.1 min (90%B to 10%B), and 35.1–45 min (10%B) as shown in Figure 3D; and Gradient Condition 3, which follows 0–2 min (20%B), 2–30 min (20%B to 75%B), 30–35 min (75%B), 35–35.1 min (75%B to 20%B), and 35.1–45 min (20%B) as depicted in Figure 3E. Following multiple optimization rounds, the chromatographic conditions outlined in Section 2.3 were finalized as the analytical method for sample detection. Under these conditions, the chromatogram achieved a minimum resolution of 1.62 between impurities and 4.95 between impurities and the main peak, both meeting detection standards (Figure 3F).

### 3.2 Method validation

The International Council for Harmonisation of Technical Requirements for Pharmaceuticals for Human Use (ICH) guideline Q2 (R1) (Group, 2005) and existing literature reports were used to guide the validation of the test method for related substances in selpercatinib (Padivitage et al., 2023).

#### 3.2.1 Specificity

Accurately pipette 10 μL of the solvent, mixed impurity solution, and system suitability solution, respectively, and inject them into the liquid chromatograph. Conduct the sample injection analysis according to the chromatographic conditions specified in Section 2.3. The chromatogram of the mixed impurity solution is shown in Figure 2B, and the retention times of selpercatinib and its respective impurities are as follows: imp-A at 10.7988 min, selpercatinib at 21.373 min, imp-B at 30.422 min, imp-C at 31.355 min, and imp- D at 32.554 min. The minimum resolution between impurities is known to be 2.15, and the minimum resolution between impurities and selpercatinib is 28.78, both of which meet the experimental requirements (>1.5).

Figure 3F presents the system suitability chromatogram. The results indicate that the minimum resolution between the main peak and adjacent impurity peaks was 4.95, the minimum resolution between known impurities and adjacent peaks was 1.72, and the minimum resolution between unknown impurities and adjacent peaks was 1.62. All these values met the experimental requirements. In summary, these results demonstrated that the method exhibited good specificity.

#### 3.2.2 Forced degradation experiments

The forced degradation tests for selpercatinib were primarily conducted under five conditions: acidic, alkaline, oxidative, high temperature, and photolytic. The specific conditions are as follows: Acidic degradation: Accurately weigh approximately 10 mg of selpercatinib into a 20 mL volumetric flask. Subsequently, measure 10 mL of methanol and dissolve the sample using ultrasound. Afterward, add 5 mL of 1 mol/L hydrochloric acid solution and heat at 70°C for 5 h. Once the reaction is complete, neutralize to neutrality using a 1 mol/L sodium hydroxide solution, and dilute with 50% methanol aqueous solution to the mark. Alkaline degradation: The procedure is similar to acidic degradation, but instead, use a 1 mol/L sodium hydroxide solution and heat at 50°C for 2 h. Oxidative Degradation: Use a 5% hydrogen peroxide solution and react at 50°C for 48 h. High Temperature Degradation: Firstly, weigh an appropriate amount of selpercatinib and place it in an oven set at 100°C for 6 days. Then, weigh 10 mg of selpercatinib and prepare a solution of 0.5 mg/mL concentration using 50% methanol solution. Photolytic degradation: Accurately measure 10 mg of selpercatinib and dissolve it to achieve a concentration of 0.5 mg/mL in a solvent composed of 50% methanol. Following preparation, subject the resultant solution to continuous illumination from an LED light source with an intensity of 4,500 lux for a duration of 30 days.

**FIGURE 4:**
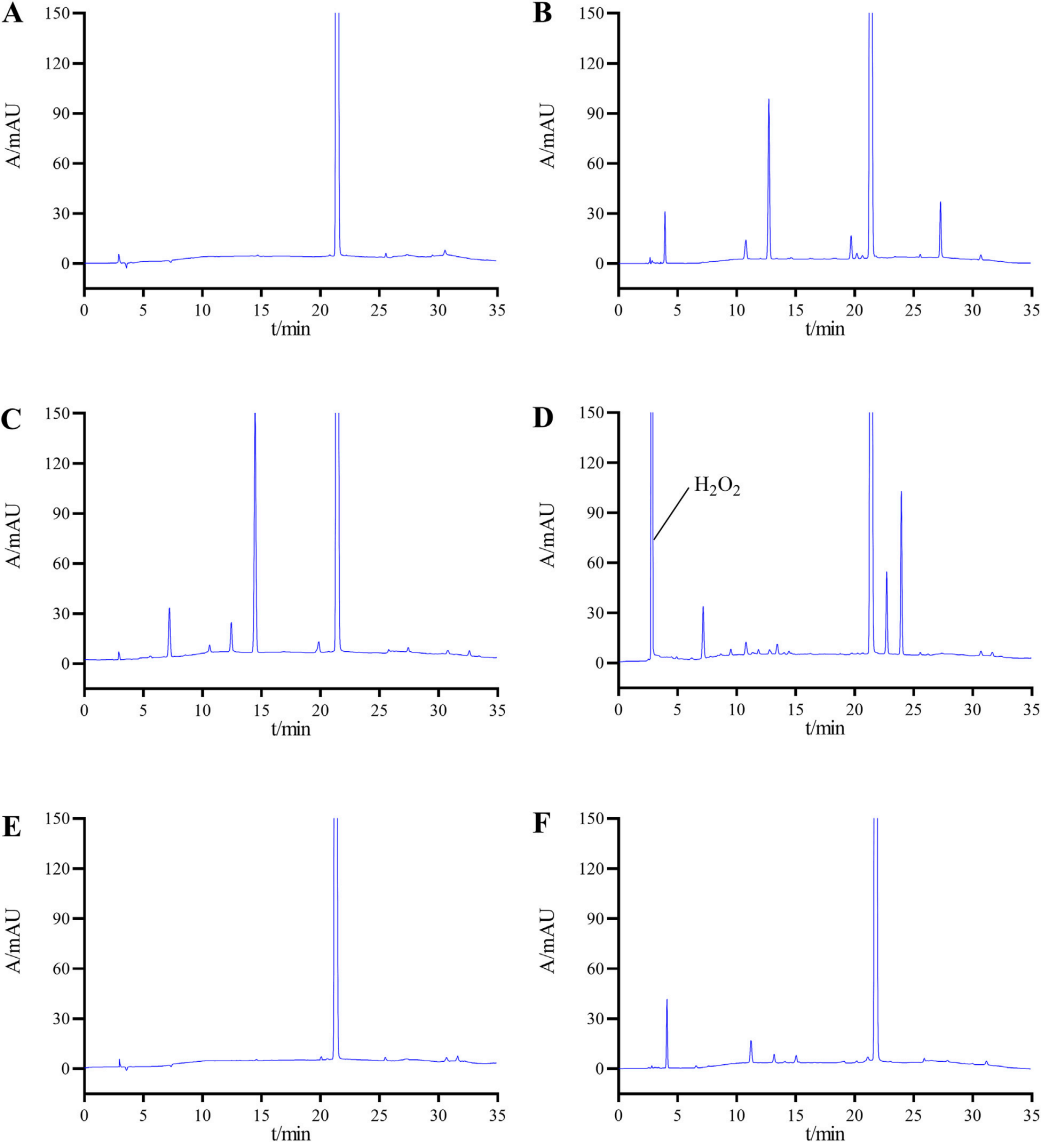
Chromatograms of selpercatinib under various degradation conditions: **(A)** undegradation; **(B)** acid-induced degradation; **(C)** base-induced degradation; **(D)** oxidative degradation; **(E)** heat degradation; **(F)** photolytic degradation.

Figure 4 displays the chromatograms from the forced degradation study of selpercatinib. Table 1 presents the stability outcomes of selpercatinib under diverse forced degradation conditions, considering impurity count, main peak content, minimum resolution between the principal component and impurities, resolution among impurities, and mass balance. The results indicate that selpercatinib exhibits remarkable stability under high temperature and light exposure conditions, whereas it is prone to degradation under acidic, alkaline and oxidative conditions. Notably, despite the varying degradation conditions, the minimum resolution between the main peak and impurities exceeds 1.5, and the minimum resolution among impurities exceeds 1.2, both of which are in compliance with the established criteria. Additionally, the mass balance rate remains within the range of 95%–105%, which is generally considered an important standard for mass balance.

**TABLE 1:** Results of forced degradation tests.

Forced degradation condition	Number of impurities (>0.03%)	Content of main peak (%)	Minimum resolution between main peak and impurities	Minimum resolution among impurities	Mass balance (%)
Undegradation	3	99.74	2.30	4.48	100
Acid degradation	9	92.45	2.65	1.89	97.53
Base degradation	8	90.08	5.83	6.86	98.22
Oxidative degradation	11	90.16	6.04	2.21	97.06
Heat degradation	4	99.12	4.89	3.66	99.37
Photolytic degradation	7	97.63	2.19	7.33	98.64

#### 3.2.3 Sensitivity

The sensitivity of the method is evaluated by determining LOD and LOQ. The stock solutions of selpercatinib and its known impurities are diluted stepwise, followed by the calculation of signal-to-noise ratios. The corresponding concentrations are determined as LOD and LOQ when the signal-to-noise ratios reach 3:1 and 10:1, respectively. The results show that the LOD for selpercatinib and its known impurities (imp-A, imp- B, imp- C, imp-D) were 0.0022, 0.0020, 0.0030, 0.0020, and 0.0030 μg/mL, respectively, while the LOQ values were 0.0065, 0.0060, 0.0085, 0.0065, and 0.0090 μg/mL, respectively.

#### 3.2.4 Stability of the solution

The stability of the selpercatinib sample solution was evaluated by analyzing samples at various time points: 0, 2, 4, 6, 8, 12, and 24 h. The primary focus was on changes in the number of impurities, the maximum content of individual impurities, and the total impurity content. The results of these analyses are detailed in Supplementary Table S1. The results indicate that after leaving the selpercatinib sample solution at room temperature for 24 h, there was no change in the number of impurities, and only minimal changes were observed in the maximum content of individual impurities and the total impurity content. This suggests that the sample remains stable at room temperature for 24 h.

Regarding the solution stability of known impurity samples, the solutions containing known impurities were placed at room temperature, and samples were analyzed at various time points. The results showed that the relative standard deviations (RSDs) of the peak areas for selpercatinib and its known impurities A, B, C, and D were 1.05%, 1.37%, 1.26%, and 1.68%, respectively, all of which were below the predetermined threshold of 2%. These results indicate that solutions containing these impurities remain stable within 24 h.

#### 3.2.5 Linearity and range

This study investigated the linearity of selpercatinib and its known impurities within a range from the LOQ to 2% of the target concentration (0.5 mg/mL was defined as 100%). The stock solutions of selpercatinib and its known impurities were diluted with 50% aqueous methanol to produce test solutions at various concentrations (0.1, 0.5, 1, 2, 5, 8, 10 μg/mL), which were then injected and analyzed according to the chromatographic conditions outlined in Section 2.3. A standard curve was generated by plotting the concentration (*X*-axis) against the peak area (*Y*-axis), and the regression equation was calculated. The linear regression equations, and correlation coefficients for selpercatinib and its known impurities were presented below: Selpercatinib: y = 58.69x-2.133, r = 0.9994; imp-A: y = 59.92x-3.084, r = 0.9988; imp-B: y = 51.55x-2.071, r = 0.9993; imp-C: y = 63.59x-6.156, r = 0.9987; imp-D: y = 50.57x-3.241, r = 0.9991. Within a range from the LOQ to 2% of the target concentration (0.5 mg/mLwas defined as 100%), all correlation coefficients exceeded 0.99.

#### 3.2.6 Precision

Take 10 μL of the mixed solution described in “2.5”and analyze it under the chromatographic conditions specified in item “2.3”. Inject the sample continuously for 6 times to evaluate the precision of the instrument. The results showed that the RSDs of the retention time for selpercatinib and its known impurities (Impurities A, B, C, D) were 0.29%, 0.16%, 0.22%, 0.19%, and 0.15%, respectively. Similarly, the RSDs of peak areas were determined to be 0.75%, 0.86%, 1.08%, 0.68%, and 1.29%, respectively. Notably, all RSD values were less than 2%, indicating good precision of the instrument.

#### 3.2.7 Repeatability

To assess the repeatability of the method, six samples containing known impurities were prepared in parallel. Analysis was then conducted according to the chromatography conditions outlined in Section 2.3. The results showed that the RSDs for impurities A, B, C, and D were 1.18%, 1.07%, 0.95%, and 1.22%, respectively. All these results were below the threshold of 2%, indicating that the method possessed good repeatability.

#### 3.2.8 Accuracy

This study tested the recovery rates of known impurities (imp-A, imp-B, imp-C, imp-D) in selpercatinib at three different concentration levels (50%, 100%, 150%), with a reference concentration of approximately 1 μg/mL set as the 100% level. To achieve the desired concentration levels, varying volumes of stock solutions containing the known impurities were added to the selpercatinib sample solutions. Triplicate samples were prepared for each concentration level, analyzed according to the chromatographic conditions described in Section 2.3, and the recovery rates were subsequently calculated. As shown in Table 2, the recovery of all known impurities fell within the range of 90%–110%, and the RSD values for these compounds were all below 2.0%. These results clearly demonstrate the high accuracy of the method employed in this study.

**TABLE 2:** Recovery of know impurities in selpercatinib.

Substance	Target level	Spiked conc. (μg/mL)	Determined conc. (μg/mL)	Recovery (%)	Average recovery rate (%)	RSD (%)
imp-A	50%	0.511	0.508	99.41	99.53	2.42
		0.511	0.528	103.33		
		0.511	0.499	97.65		
	100%	1.021	1.052	103.04		
		1.021	0.992	97.16		
		1.021	1.032	101.08		
	150%	1.532	1.516	98.96		
		1.532	1.492	97.39		
		1.532	1.498	97.78		
imp-B	50%	0.508	0.495	97.44	99.29	2.15
		0.508	0.512	100.79		
		0.508	0.499	98.23		
	100%	1.016	0.998	98.23		
		1.016	1.029	101.28		
		1.016	0.986	97.05		
	150%	1.524	1.506	98.82		
		1.524	1.497	98.23		
		1.524	1.578	103.54		
imp-C	50%	0.506	0.495	97.83	97.40	1.84
		0.506	0.487	96.25		
		0.506	0.511	100.99		
	100%	1.012	0.995	98.32		
		1.012	0.975	96.34		
		1.012	0.968	95.65		
	150%	1.518	1.455	95.85		
		1.518	1.462	96.31		
		1.518	1.504	99.08		
imp-D	50%	0.517	0.523	101.16	98.11	2.09
		0.517	0.508	98.26		
		0.517	0.493	95.36		
	100%	1.034	1.008	97.49		
		1.034	0.992	95.94		
		1.034	1.026	99.23		
	150%	1.551	1.493	96.26		
		1.551	1.529	98.58		
		1.551	1.562	100.71		

#### 3.2.9 Robustness

The robustness of the method was evaluated by small but deliberate changes to HPLC parameters including column temperature (±5°C), flow rate (±0.1 mL/min), detector wavelength (±5 nm), %B at initial change (±1%) and chromatographic columns from different manufacturers.

The minimum resolution between selpercatinib and known impurities, as well as adjacent impurities, exceeded 1.5, while the minimum resolution between unknown impurities was greater than 1.2. Both of these values fulfilled the validation requirements. Additionally, there was no significant change in the number and content of impurities. This results indicated that the method developed in this study demonstrated good robustness.

## 4 Conclusion

In this study, we developed a HPLC method specifically for detecting substances associated with the non-small cell lung cancer treatment drug selpercatinib, including process impurities and degradation impurities. Additionally, we conducted a comprehensive validation of this detection method, which demonstrated excellent performance in specificity, sensitivity, linearity, precision, accuracy and robustness. Therefore, the chromatographic method established in this study is of great significance for ensuring the quality of selpercatinib and enhancing the safety and efficacy of its use.

## Data Availability

The original contributions presented in the study are included in the article/Supplementary Material, further inquiries can be directed to the corresponding authors.
