# Supplementary material for: Research on detection methods of related substances and degradation products of the antitumor drug selpercatinib
Source: Front Chem. 2025 Jan 13;12:1534132. doi: 10.3389/fchem.2024.1534132 (PMC11770003; doi:10.3389/fchem.2024.1534132)
Supplement: Supplementary file 1 [file DataSheet1.docx]

Supplementary Material


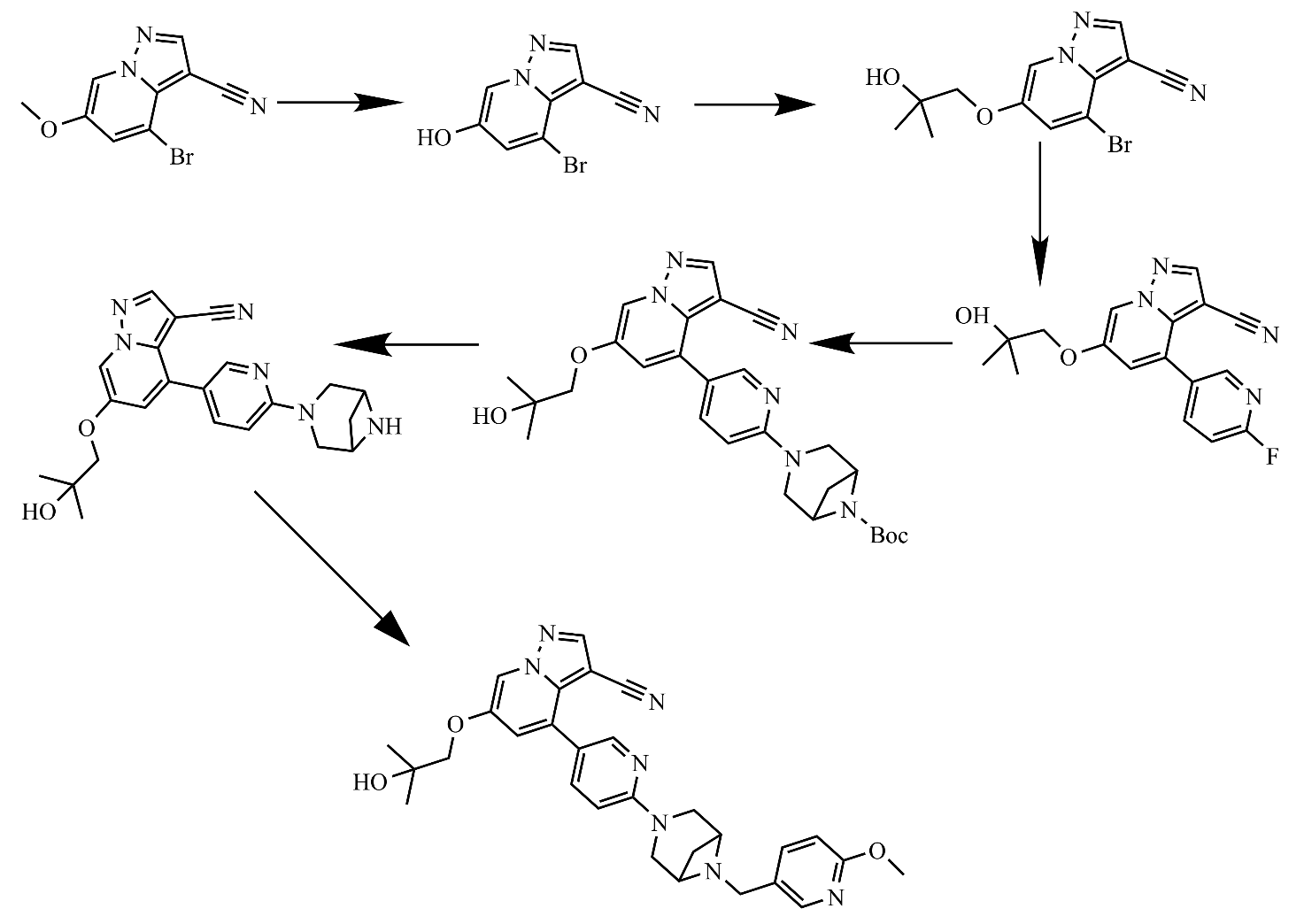


**Figure S1** Synthetic route of selpercatinib

**Table S1** Test results of selpercatinib sample solution stability

| **Time/h** | **Number of impurities** | **Content of maximum single impurity (%)** | **Content of total impurities (%)** |
| --- | --- | --- | --- |
| 0 | 3 | 0.11 | 0.26 |
| 2 | 3 | 0.12 | 0.27 |
| 4 | 3 | 0.11 | 0.26 |
| 6 | 3 | 0.13 | 0.28 |
| 8 | 3 | 0.12 | 0.28 |
| 12 | 3 | 0.13 | 0.29 |
| 24 | 3 | 0.14 | 0.31 |
